# In vitro micropropagation of *Aloe elegans* Tod. using offshoot cuttings

**DOI:** 10.1186/s13104-023-06490-0

**Published:** 2023-09-12

**Authors:** Mebrahtom Welehaweria, Desta Berhe Sbhatu

**Affiliations:** 1 Department of Biology, College of Natural and Computational Sciences, Mekelle University, PO Box 231, Mekelle, Ethiopia; 2 Department of Biological and Chemical Engineering, Mekelle Institute of Technology, Mekelle University, PO Box 1632, Mekelle, Ethiopia

**Keywords:** Acclimatization, *Aloe elegans*, Initiation, Micropropagation, Rooting, Shooting

## Abstract

**Objective:**

*Aloe elegans* Tod. is an ecologically, environmentally, medicinally, and commercially useful aloe species in Ethiopia and Eritrea. Unfortunately, it is highly threatened due to industrial and urban expansion and traditional mining and agricultural activities. As a consequence, it is included in the IUCN List of Threatened Species since 2013. The plant is getting thinly populated in many parts of the Tigrai floristic region since it is being exploited for traditional and commercial purposes. Therefore, this study was aimed to develop a reproducible, large-scale micropropagation protocol using offshoot cuttings in Murashige and Skoog (MS) media enriched with plant growth regulators (PGRs).

**Results:**

Sterilized explants cultured in full-strength MS media enriched with 0.25 mg/L benzyl amino purine (BAP) and 0.10 mg/L naphthaleneacetic acid (NAA) resulted in 100% healthy and live (i.e., initiated) explants after four weeks of initiation study. Unsupplemented initiation media (control) yielded only 14.3% initiated explants. The initiated explants were tested for their shooting response to produce microshoots by incubating in different concentrations and combinations of BAP and NAA for four weeks. Fewer days to shooting (13.0 ± 1.0 days), higher mean shoot number (5.0 ± 1.0), and higher mean shoot length (9.20 ± 2.35 cm) were observed with 1.0/0.50, 1.0/0.25, and 1.0 /0.50 mg/L BAP/NAA combinations, respectively. The rooting responses of the microshoots toward producing plantlets were also tested by incubating them in half-strength MS media enriched with different concentrations of NAA and indole-3-butyric acid (IBA) for four weeks. Fewer mean days to rooting (12.0 ± 1.0 days), higher mean root number (8.0 ± 4.0), and higher mean root length (7.53 ± 3.03 cm) were observed in MS media enriched with 0.75, 0.75, and 1.25 mg/L IBA, respectively. The responses of *A. elegans* plantlets to primary (in greenhouse) and secondary (in nursery shade and direct sunlight) acclimatization in coco peat, composted soil, and manured soil media were high – with survival percentages of 87.5–97.8% in three to four weeks.

## Introduction

*Aloe* L. (Aloaceae) comprises the most familiar and useful succulent flowering plants. Ethiopia and Eritrea are home to 50 species of *Aloe* of which 31 being endemic. They grow in various altitudes stretching from sea level at the Red Sea coast of Massawa, Eritrea (*A. eumassawana* Carter, Gilbert & Sebsebe) to about 3,500 m at Ankober of Showa, Ethiopia (*A. ankoberensis* Gilbert & Sebsebe) exhibiting high degree of endemism [1, 2]. *A. elegans* – a wild species not known anywhere else – grows in rocky slopes, mostly on sandstone (limestone), in evergreen bushlands, and wooded grasslands between 1,500 and 2,400 m in Tigrai, Wollo, Gojjam, and Showa floristic regions in Ethiopia and in Eritrea [1].

Despite their ecological, environmental, traditional (in traditional medicine), and commercial benefits, aloes are highly threatened due to industrial and urban expansion and traditional mining and agricultural activities and developments. They are highly endemic and naturally restricted to small geographical areas. Moreover, they propagate vegetatively very slowly in their natural environments [1]. Therefore, nearly all species of *Aloe*, including the Ethiopian aloes, are listed in the CITES (Convention on the International Trade in Endangered Species of Wild Fauna and Flora) Appendix II to prohibit the commercial exploitation of the plants from their wild stands [3, 4].

Preliminary studies on its ethnobotany and phytochemistry of its gel have showed that *A. elegans* has high potential as sources of cosmetic and toiletry, medicinal, and pharmaceutical products [3–5]. However, we have observed the plant in many parts of the Tigrai floristic region as thinly populated. For this reason, it is included in the IUCN List of Threatened Species since 2013 [6]. Commercial exploitation of the plant will, therefore, require large-scale cultivation. In line with this rationale, a reproducible protocol for its in vitro propagation is developed to lay a technical foundation for its large-scale cultivation.

## Materials and methods

### Collection and sterilization of explants

Explants of *A. elegans* were collected from wild stands. Collection of biological materials from the wild by native (Ethiopian) researchers for research and development purposes is granted by Article 15, Clause 1 of the Access to Genetic Resources and Community Knowledge, and Community Rights Proclamation of Ethiopia (Proclamation No. 482/2006) without requiring written permission. Specimen of the plant was identified by the second author and verified by a curator in the Aklilu Lemma Institute of Pathobiology, Addis Ababa University, Ethiopia; and a voucher specimen was deposited at the Endod and Other Medicinal Plants Research Unit of the Institute (ID: DB003/19). Vigorous and healthy looking mother plants were located. Then, offshoots were carefully dug out from the base of the mother plants without causing mechanical damages and contaminations. Specimens were trimmed to 1.5–2.0 cm long explants for sterilization. Primary and secondary sterilizations of the explants were carried out according to standard procedures by using: tap, distilled and sterilized distilled water; Tween-20 and soap solution; aqueous solution (of 0.25% rocide, 0.25% ridomile and 0.25% bayleton); 5% v/v of NaOCl; and HgCl_2_ (0.1% w/v aqueous solution) [7–9].

### Micropropagation experiments

The standard procedure of Murashige and Skoog (MS) [10] was employed to prepare sterile growth media enriched with three plant growth regulators (PGRs), namely benzyl amino purine (BAP), naphthaleneacetic acid (NAA), and indole-3-butyric acid (IBA). Initiation and shooting experiments were carried out by using full-strength MS media while rooting experiment was conducted using half-strength media. Firstly, shoot initiation experiment was conducted by inoculating 105 sterilized explants distributed in three groups in full-strength MS media enriched with 0.25 mg/L BAP and 0.10 mg/L NAA in 300 mL magenta culture bottles. Thirty-five (35) additional explants were inoculated into full-strength MS media without BAP and NAA. Each bottle has one explant. Explants were incubated in growth room for four weeks for initiation. Secondly, shooting experiment was carried out using four shooting media enriched with 1.0 + 0.25, 1.0 + 0.50, 1.50 + 0.25 and 2.0 + 0.25 mg/L BAP and NAA, respectively, and one non-enriched media (control) in four replicates. Initiated explants were harvested from the initiation media and one initiated explant was cultured in each culture bottle. The cultured explants were incubated for four weeks to produce viable microshoot. Thirdly, rooting experiment was conducted by inoculating 2–3 cm long viable microshoots. The microshoots were harvested from the shooting media and were transferred into culture bottles containing half strength MS media. Six enriched rooting media (i.e., 0.75, 1.25, and 1.50 mg/L of NAA and 0.75, 1.25, and 1.50 IBA) and one non-enriched media (control) were prepared in culture bottles in five replicates. One microshoot was cultured in each bottle and all microshoot-containing bottles were incubated for four weeks. Rooting was operationally defined as the emergence of ≤ 1 cm piece of root. Rooting shoots were kept in the growth room until many of the shoots grow to ≥ 5 cm long plantlets. Initiation, shooting, and rooting experiments were carried out in growth room racks at 25 ± 0.5 °C temperature under fluorescent tube light, 16 h photoperiod, and 2,000–2,500 lx light intensity.

### Acclimatization study

Plantlets (46) with well-developed roots were harvested from the rooting media after four weeks; and were carefully washed with running tap water to remove any traces of agar and sucrose. Then, they were soaked in hot water (ca. 40 °C) for about 5 min to remove any oily stuff from the root surface that would hamper water and nutrient absorption during acclimatization. Primary acclimatization experiment was carried out in greenhouse. The plantlets were planted in Pro tray with cocopeat and kept in greenhouse for three-week primary acclimatization. The microclimate of the greenhouse was manipulated to progress from high relative humidity (80–90), low temperature (25 ± 2 °C), and low light intensity (1,200 lx) through medium relative humidity (70–80), medium temperature (26 ± 2 °C), and medium light intensity (2,500 lx) to low relative humidity (60–70), high temperature (27 ± 2 °C), and high light intensity (5,000 lx) over the three weeks. Secondary acclimatization experiment was conducted under nursery shade and direct sunlight. Fourty one (41) plantlets with similar size and vigor that survived primary acclimatization were divided into four groups of 13, 10, 10 and 8 plantlets and transplanted into soil media. Two types of soil media composed of sand, soil, and compost (i.e., composted soil media) and sand, soil, and manure (i.e., manured soil media) at 1:1:1 proportion were prepared and filled in polyethylene bags (height 15 cm; diameter 9 cm). Two groups of plantlets were planted in the composted soil and the other two were planted in the manured soil. One group of plantlets in each soil medium was placed under nursery shade, and the other group of plantlets was placed under direct sunlight. The plantlets were kept for three weeks by watering daily with no nutritional supplements.

### Data collection and analyses

Quantitative data including: number of explants surviving initiation experiment, number of days to shoot and root emergence, number of shoots and roots per bottle, length of shoots and roots per plantlet, and survival rate of plantlets after acclimatization were recorded and organized for analyses. Data of days to shooting and rooting were collected by observing the incubated microshoots every two days. Data on the number and length of shoots and roots were collected after the shooting and rooting experiments were concluded, respectively. Qualitative observations were made to support the quantitative data. Data were analyzed through analysis of variance (ANOVA) using the statistical package for social science (SPSS Version 20). Comparisons of mean (± SD) values were made at *a priori* set significance level of *p* ≤ 0.05.

## Results and discussion

### Initiation response

The initiation experiment resulted in 110 (78.6%) 1.5 to 2.0 cm long clean and viable explants (i.e., initiated explants). All explants incubated in enriched media were survived (100%) but only 5 out of the 35 (14.3%) plantlets incubated in unsupplemented media were survived. Many studies have succeeded in producing initiated explants with offshoots using a combination of different concentrations of BAP and NAA [7, 11–13]. In vitro propagation media enriched with 0.20/0.20 mg/L, 4.0/0.20 mg/L, 0.50/0.50 mg/L, and 0.20/0.20 mg/L BAP/NAA have been effective for initiating growth in *A. percrassa* Tod. [7], *A. vera* L. [11], *A. trichosantha* Berger [12], and *A. adigratana* Reynolds [13], respectively. BAP and NAA are often used for initiating explants of many aloe species. However, establishing the right combinations and concentrations for the best initiation response requires further enquiry.

### Shooting responses

**Fig. 1:**
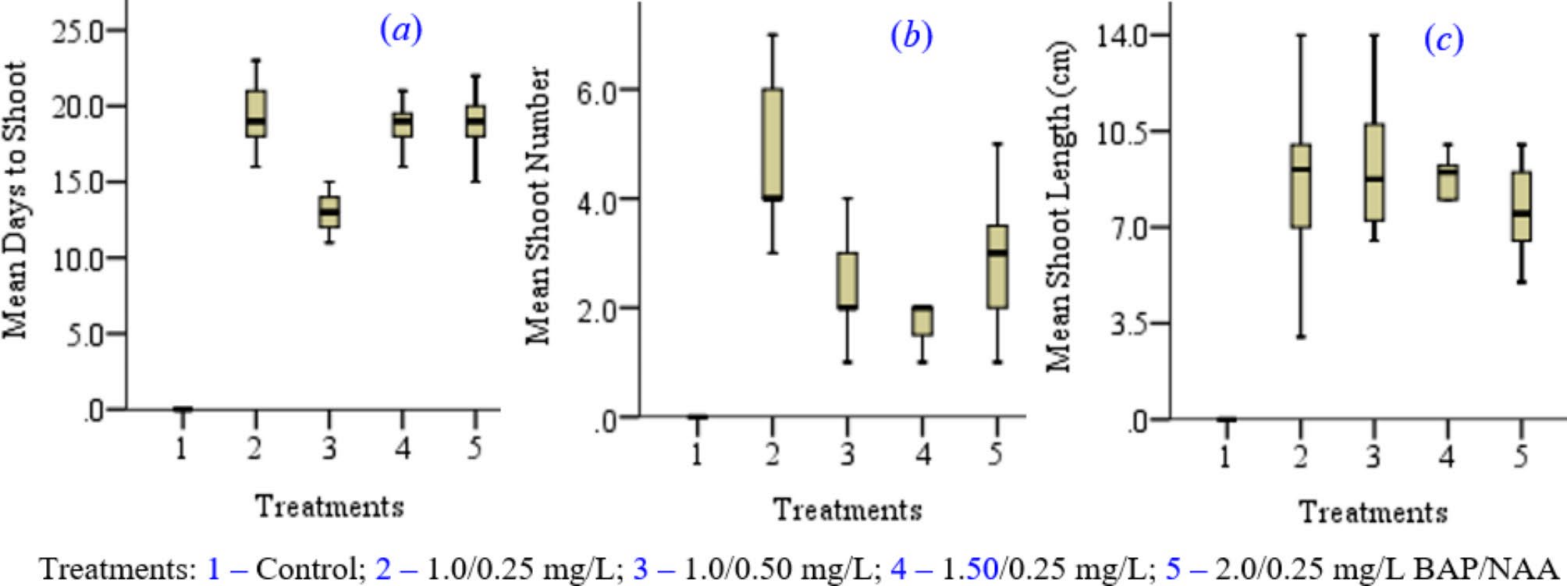
Different concentrations of BAP and constant concentration of NAA on shooting of *A. elegans* Tod

Results of ANOVA indicated that the mean (±SD) number of days to shooting, shoot number, and shoot length were statistically significantly different among the treatments (*p* ≤ 0.05) (Fig. 1(*a*), (*b*) (*c*)).

Explants of *A. elegans* cultured in MS media enriched with different concentrations and combinations of BAP and NAA took 13.0 ± 1.0 to 19.0 ± 2.0 days on average to shoot. These results indicated that 13.0 ± 1.0 days to shooting (in MS medium enriched with 1.0 mg/L BAP + 0.50 mg/L NAA) was statistically significantly shorter (*p* ≤ 0.05) (Fig. 1(*a*)). Many other studies have reported shooting in *A. adigratana* Reynolds, *A. percrassa* Tod., *A. trichosantha* Berger, and *A. vera* L. within two weeks in MS media enriched with 1.0 mg/L BAP in combination with auxins (such as IAA and NAA) [7, 12–14].

**Fig. 2:**
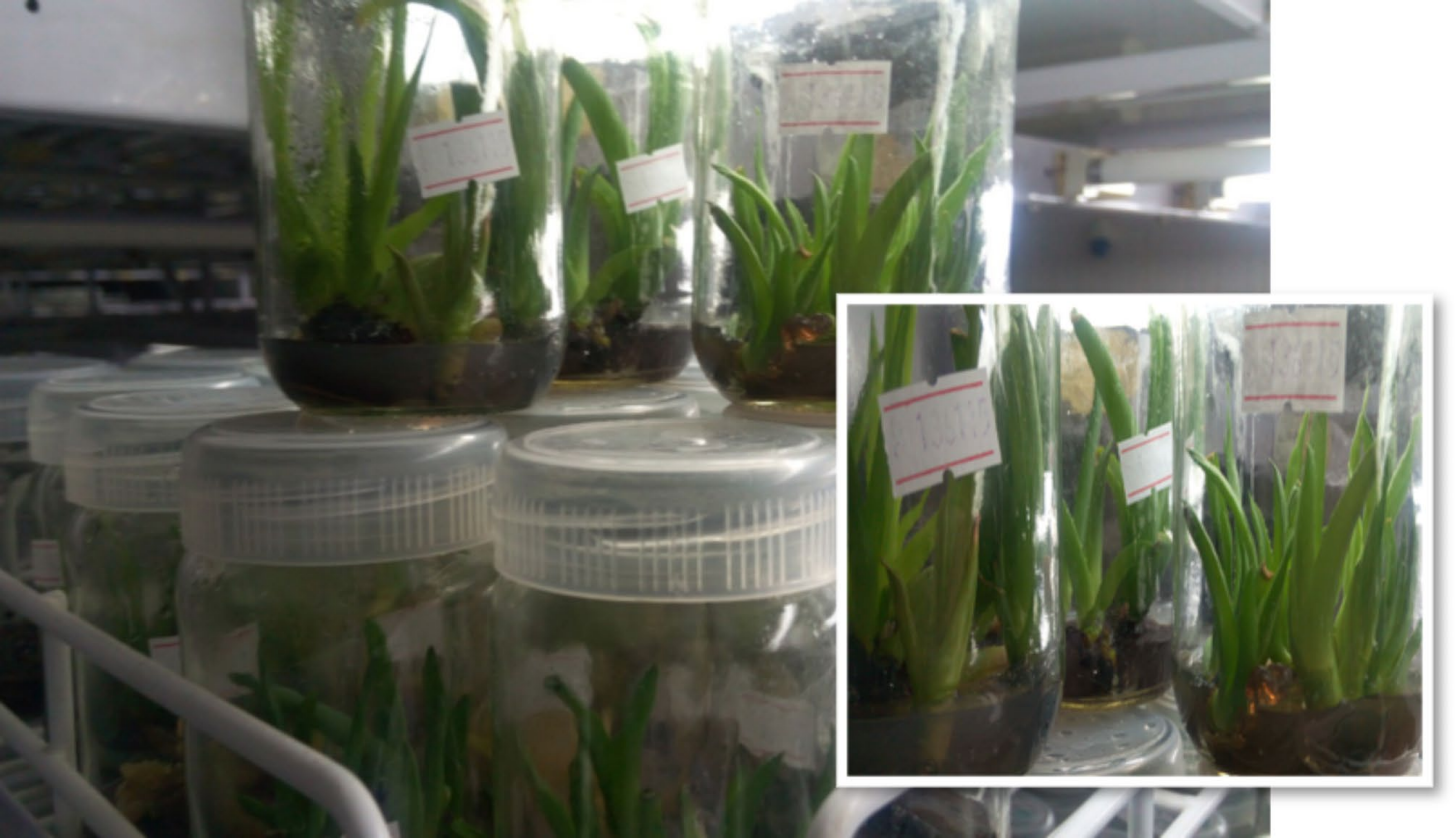
Shooting response of *A. elegans* Tod. with different BAP/NAA supplementations. (Shoots in the smaller frame were cultured in MS medium with 1.0/0.25 mg/L BAP/NAA supplement)

MS media enriched with BAP and NAA have produced 2.0 ± 1.0 to 5.0 ± 1.0 shoots within three weeks. Mean shoot number of 5.0 ± 1.0 per plantlet cultured in MS media enriched with 1.0 mg/L BAP + 0.25 mg/L NAA was significantly higher than the mean values observed in other treatments (*p* ≤ 0.05) (Figs. 1(*b*) and 2). Combination of BAP and NAA at 1.0 mg/L BAP and 0.50 mg/L NAA, respectively, has resulted in significantly higher mean shoot numbers in many aloes species [7, 13, 15, 16]. However, other studies have reported higher mean shoot number per explant at higher concentrations of BAP [8, 12, 17, 18].

Explants cultured in MS media supplemented with various concentrations and combinations of BAP and NAA have produced shoots with mean length ranging from 7.62 ± 1.60 cm (cultured in MS medium supplemented with 2.0 mg/L BAP + 0.25 mg/L NAA) to 9.20 ± 2.35 cm (cultured in MS medium supplemented with 1.0 mg/L BAP + 0.50 mg/L NAA). The mean shoot length of 9.20 ± 2.35 cm was significantly higher than the rest of the mean values (*p* ≤ 0.05). No shooting response was observed in the control (Fig. 1(*c*)). Many studies with other species have reported the highest mean shoot length in plantlets cultured in MS media supplemented with 1.0 mg/L BAP in combination with 0.5 mg/L NAA as compared to other combinations of PGR supplements [7, 12, 13, 18].

High mean shoot lengths were also reported with lower (0.50 mg/L) [12, 19, 20] and higher (2.00 to 4.00 mg/L) BAP in combination with 0.50 mg/L NAA [19–21]. This research and so many other studies have generally shown that the supplementation of low (0.50 mg/L) through medium (2.0 mg/L) to high (4.0 mg/L) BAP in combination with 0.5 mg/L of NAA produce microshoots with high mean length. When all the variables of good shooting response are considered, 1.0–1.50 mg/L BAP in combination with 0.25–0.50 mg/L NAA lead to better micropropagation performance in Ethiopian aloes [7, 12, 13].

### Rooting responses

The ANOVA results showed that means of days to rooting, number of roots, and length of roots were statistically significantly different between the different treatments (*p* ≤ 0.05) (Table 1).

**Table 1:** Different concentrations of NAA and IBA on rooting responses of *A. elegans* Tod

PGRs	Concentration (mg/L)	Mean (SD) values		
		No. of days to rooting	Root number	Root length, cm
Control	0.00	–	–	–
NAA	0.75	17.0 (3.0)^a^	7.0 (4.0)^b^	5.2 (2.3)^b^
	1.25	17.0 (2.0)^a^	7.0 (4.0)^b^	3.8 (2.2)^a^
	1.50	17.0 (3.0)^a^	4.0 (3.0)^a^	3.6 (2.4)^a^
IBA	0.75	12.0 (1.0)^b^	8.0 (4.0)^c^	4.4 (2.0)^a^
	1.25	19.0 (2.0)^a^	4.0 (2.0)^a^	7.5 (3.0)^c^
	1.50	17.0 (1.0)^a^	7.0 (4.0)^b^	7.4 (2.2)^c^
Mean		14.72	5.48	4.14
CV%		45.00	76.00	72.00
LSD		0.07	0.00	0.03

Means in the same column with different letters are statistically significantly different at *p* ≤ 0.05; CV: Coefficient of variance (%); LSD: Least significant different

Microshoots grown in half-strength MS media supplemented with three concentrations of NAA and IBA rooted in 12.00 ± 1.00 to 19.00 ± 2.00 days. Rooting medium supplemented with 0.75 mg/L IBA has resulted in rooting in 12.00 ± 1.00 days – statistically significantly less than all other treatments (*p* ≤ 0.05). Auxins (especially IBA and NAA) are most commonly used PGRs for rooting of shoots [7, 12, 13, 16, 22–27]. Many studies have reported rooting of aloe microshoots within one and three weeks in MS media supplemented with 1.0 to 2.0 mg/L of IBA [7, 13, 25]. Similarly, rooting media supplemented with 0.50–1.50 mg/L NAA have caused rooting responses in less than 15 days [7, 12, 13, 22]. Many studies have shown that aloe shoots develop roots in 9–30 days but it is hard to draw clear pattern when PGRs’ concentrations increase or decrease [7, 12, 13].

The mean root number of the rooted shoots, technically known as plantlets, ranged from 4.00 ± 2.00 to 8.00 ± 4.00. Half-strength MS medium enriched with 0.75 mg/L IBA has produced plantlets with significantly higher mean root number (8.00 ± 4.00; *p* ≤ 0.05) (Table 1). *A. adigratana* Reynolds shoots cultured in rooting media enriched with 0.50 to 1.50 mg/L IBA have produced 8.4 to 11 roots per shoot [13]. IBA (1.0 mg/L) in combination with activated charcoal (500 mg/L) has resulted in higher mean root number (5.42) per explant in *A. barbadensis* Mill. [17]. The present study has also shown that shoots cultured in MS media enriched with 0.75 and 1.25 mg/L NAA produced 7.0 ± 4.0 roots per shoot (*p* ≤ 0.05). A study with *A. percrassa* Tod. has resulted in higher mean root number in shoots cultured in MS media with low concentration of NAA supplements – decreasing with increasing concentration from 8.4 in unsupplemented medium to 3.4 with 1.50 mg/L NAA [7]. A similar trend was observed in *A. adigratana* Reynolds [13]. Another study on *A. barbadensis* has recorded the highest mean number of roots per shoot (4.8 ± 0.53) when cultured with 0.50 mg/L NAA supplementation [27]. Another study has also reported a better rooting response in *A. indica* L. with 0.50 mg/L NAA [26]. On the contrary, a research with *A. trichosantha* Berger showed that the mean number of roots increases with increasing NAA supplements from 0.50 to 1.0 mg/L [12].

Similarly, the mean root length of shoots grown in MS media supplemented with NAA and IBA ranged from 3.62 ± 2.37 to 7.53 ± 3.03 cm. Higher IBA concentrations have resulted in higher mean root lengths (Table 1). On the other hand, higher mean root lengths were observed with increasing concentration of NAA from 0.75 to 1.50 mg/L. In a study with *A. adigratana* Reynolds, mean root length decreased with increasing NAA concentration from 0.50 to 1.50 mg/L while the opposite was observed with IBA [13]. In one study with *A. trichosantha* Berger, it was shown that the mean root length decreases as NAA concentration decreases from 0.25 to 1.50 mg/L [12]. Other researchers have observed similar patterns with unsupplemented and low NAA supplemented (0.50 mg/L) media producing the highest mean root lengths of up to 6.0–7.0 cm [7, 11, 28]. In the future, optimization of rooting media for *A. elegans* shoot culture should focus on lower NAA and higher IBA supplementations.

### Acclimatization

Fourty five (97.8%) of *A. elegans* plantlets exposed to primary acclimatization in the greenhouse have survived. Many studies on aloes have reported high primary acclimatization rates – e.g., 100% with *A. trichosantha* Berger [12], 96.6–100% with *A. adigratana* Reynolds [13], 94–100% with *A. percrassa* Tod. [7], 95–100% with *A. vera* L. [14, 18], and 95% with *A. barbadensis* Mill. [17]. Acclimatization media affect the success of acclimatization of the delicate heterotrophic plantlets. Coco peat, composted and manured soil, perlite and vermiculite soil, and other media are preferred because they facilitate drainage and aeration. Primary and secondary acclimatization trials with coco peat often yield up to 100% survival rate [7, 12, 13].

The secondary acclimatization experiment has yielded 87.5 to 92.3% survival rate; with a single plantlet died from each group. Aloes are hardy plants capable of withstanding harsh environmental conditions. It is, thus, natural for aloe species to easily acclimatize. The present study has observed similar survival rates of plantlets subjected to secondary acclimatization with no difference due to rooting media or microclimate. The secondary acclimatization tests under shaded nursery or direct sunlight did not make a difference. We have observed that the death of the single plantlet in each treatment was not linked to physiologic or anatomic reasons but due to physical damages. Regardless of the planting soil media or light conditions (nursery shade or direct sunlight), high survival of aloe plantlets subjected to secondary acclimatization is common [7, 12–14, 18]. Healthy and undamaged plantlets often survive and grow profusely.

## Conclusion

This study has shown that initiation and shooting of *A. elegans* explants required PGRs-supplemented MS media. Explants of the species responded to full-strength MS shooting media enriched with 1.0/0.50 mg/L BAP/NAA in less than two weeks. MS shooting media supplemented with 1.0 mg/L BAP in combination with 0.25 to 0.50 mg/L NAA were effective in yielding the highest mean shoot number and length. Likewise, well-developed shoots of this aloe produced roots in less than two weeks in half-strength MS media enriched with 0.75 mg/L IBA. IBA supplementation of rooting media at 0.75 mg/L has caused the highest mean number and length of roots. Healthy and vigor plantlets of the species have survived primary and secondary acclimatization treatments under different microclimates and planting media. These findings will serve as basis for developing better and refined in vitro propagation protocol for the species. Therefore, further researches can concentrate on having many PGRs supplementations and combinations.

### Limitations

The acclimatization study involved relatively fewer plantlets because the plantlets selected for the study were supposed to have comparable size and vigor.

## Data Availability

The datasets used and/or analyzed during the current study are available from the corresponding author on reasonable request.

## Abbreviations

ANOVA: Analysis of variance
BAP: Benzyl amino purine
CITES: Convention on the International Trade in Endangered Species
CV: Coefficient of variance
HgCl: Mercuric chloride
IBA: Indole-3-butyric acid
IUCN: International Union for Conservation of Nature
LSD: Least significant different
mg/L: Milligram per Liter
MS: Murashige and Skoog
NAA: Naphthaleneacetic acid
NaOCl: Sodium hypochloride
PGRs: Plant growth regulators
SD: Standard deviation
SPSS: Statistical package for social science
